# Precursors to non-invasive clinical dengue screening: Multivariate signature analysis of in-vivo diffuse skin reflectance spectroscopy on febrile patients in Malaysia

**DOI:** 10.1371/journal.pone.0228923

**Published:** 2020-04-01

**Authors:** Abdul Halim Poh, Faisal Rafiq Mahamd Adikan, Mahmoud Moghavvemi, Sharifah Faridah Syed Omar, Khadijah Poh, Mohamad Badrol Hisyam Mahyuddin, Grace Yan, Mohammad Aizuddin Azizah Ariffin, Sulaiman Wadi Harun

**Affiliations:** 1 Department of Electrical Engineering, Faculty of Engineering, University of Malaya, Kuala Lumpur, Malaysia; 2 Integrated Lightwave Research Group, Faculty of Engineering, University of Malaya, Kuala Lumpur, Malaysia; 3 Center of Research for Applied Electronics (CRAE), Faculty of Engineering, University of Malaya, Kuala Lumpur, Malaysia; 4 University of Science and Culture, Tehran, Iran; 5 Faculty of Medicine, University of Malaya, Kuala Lumpur, Malaysia; 6 Infectious Diseases and Medicine, University of Malaya, Kuala Lumpur, Malaysia; 7 Photonics Engineering, Faculty of Engineering, University of Malaya, Kuala Lumpur, Malaysia; University of Colombo Faculty of Medicine, SRI LANKA

## Abstract

Dengue diagnostics have come a long way. Attempts at breaking away from lab-oriented dengue detection, such as NS1 antigen, IgM or IgG antibodies detection have extensively received numerous coverage. As a result, rapid detection tests (RDTs) have started to gain inroads in medical practice. Rapid detection tests notwithstanding, analysis of blood serum is still a relatively complicated task. This includes the necessity of phlebotomy, centrifugation for blood serum, and other reagent-based tests. Therefore, a non-invasive method of dengue detection was considered. In this study, we present the utility of diffuse reflectance skin spectroscopy (bandwidth of 200-2500nm) on the forearm during the triaging period for dengue screening potential. This is performed with multivariate analysis of 240 triaged febrile/suspected dengue patients. The data is then scrutinized for its clinical validity to be included as either confirmed or probable dengue, or a control group. Based on discriminant analysis of several data normalization models, we can predict the patients’ clinical dengue-positivity at ranges of accuracy between ~93–98% depending on mode of the data, with a probably optimal sensitivity and specificity to the clinical diagnosis of ~89% and ~100% respectively. From the outcomes of this study, we recommend further trials with cautious optimism. With these findings, it is hoped that the elusive non-invasive detection of tropical diseases may gain platform in the near future.

## I. Introduction

Dengue fever (DF), caused by dengue virus (DENV) is one of the common causes of acute febrile illness in Southeast Asia. In Southeast Asia, it afflicts 50–100 million every year, with a potential exposure to 3 billion people globally [1]. DENV is transmitted mainly via *Aedes Aegypti* mosquitoes, infected by feeding on other humans or animals in the viremic stage [2]. Upon biting a victim, it takes 4–10 days for the symptoms to appear, which primarily starts with a fever, usually exacerbating within a short time. Other symptoms may appear depending on the severity of the infection, including vomiting, nausea and others, although the majority of the victims are asymptomatic [1]. However, the morbid reputation of dengue infections is largely credited to the aftereffects of a second dengue infection. In this case, during defervescence or critical phase, severe symptoms would include plasma leakage which cause hypovolemic shock and death in some cases. [2, 3]. As symptoms of dengue fever are non-specific, clinicians rely on other diagnostic tools to help them make an accurate diagnosis at the bedside. However, laboratory base and point-of-care (POC) diagnostics may not be readily available and will need to be interpreted with reference to the clinical timelines.

### Clinical and laboratory confirmation of dengue

During the initial phase of the illness for infected patients (Day 1–7), DENV entry causes host cells to release polyprotein precursors, subsequently cleaved into structural and non-structural proteins, with NS1 antigen as one of the latter [4]. The seroconversion (typically Day 5 onwards) then occurs resulting in another key product of the immunological response, namely the IgM followed by IgG antibodies. Normally, detection for these components are part of the diagnostics process. These proteins are usually detected via variants of enzyme-linked immunosorbent assays (ELISA) [5]. If required, dengue will further be confirmed with the golden standard combination of reverse-transcriptase polymerase chain reaction (RT-PCR) or Haemagglutination Inhibition (HI) assays [5, 6]. However, its complexity, time consumption and overhead required for personnel makes it unfeasible for widespread use. Laboratory-confirmed results, including the binary readings of the NS1 antigens and IgM-IgG proteins will be subject to further scrutiny before a diagnosis is provided. It is important for the clinicians to interpret the results of these tests in relation to the clinical timelines and also the possibility of false positive and false negative results. This is performed by cohort sessions with infected patients, ultimately concluded after discriminating presumptive and confirmed cases [7]. An accurate diagnosis can often take up to 3–4 days with cohort patient care [8].

### Rapid detection methods and arising issues

In the realm of photonics, several in-situ dengue diagnostics have been developed recently. A label free detection using multimode-tapered fiber, which is bio-functionalized with glycoprotein specifically reacts to the NS1 and IgG proteins under two minutes [9]. The use of surface plasmon resonance (SPR) method to examine the dynamics of IgM antigens has also gained traction recently [10]. The use of microfluidics combined with fluorescence imaging for rapid diagnostics of febrile diseases including Zika, Chikugunya and Dengue (types 1 and 3) also has been reported recently. This was achieved via pinprick on the finger and application of the blood on the chip followed by a smartphone-based image processing for an in-situ classification, capable of diagnostics within 60 minutes [11]. Perhaps a relatively much simpler method, there also have been attempts at linking spectroscopic analysis with dengue. One of these techniques employs the use of lateral flow assays (LFA) by administering the blood sera on a paper-based immunoassays followed by excitation using a He-Ne laser on the sample [12]. The recent use of Raman spectroscopy on human sera with NS1/IgM positive results has achieved an optimistic outcome [13, 14]. However, despite the growing number of rapid detection techniques on dengue, the economic impact on society it has currently faces questioning [15]. A corpus on various methods of dengue diagnostics and its limitation has also been summarized, highlighting in many cases issues pertaining to its cost-effectiveness and complexity [16, 17].

### Is it time to take it further?

All of the above new dengue diagnostics methods, require blood to be drawn from patients with dengue; probably one of the biggest stumbling block to take these new methods further. Perhaps it is time to circumvent the technicalities of rapid detection schemes by considering non-invasive methods. Other than a point-of-care (POC) application, this results in bypassing the procedures and time/financial costs linked to RT-PCR, HI Assays and other ELISA methods as a whole, including phlebotomy, labeling, centrifugation for the blood sera, and others. The possible complications arising from each step would also be averted.

Although the visible range (390–700 nm) is discernible to the human eye, a non-invasive detection of oxy- and deoxygenated blood occurs within the same range as with a spectrometry setup, a feat not achievable by mere human observation. This demonstrates the potential for spectrometric methods to unravel detail from dengue patients. Established since 1981, the depths of penetration of optical waves through the human skin can go from 2 to 2200 microns [18], lending credence to the possible consideration of straightforward non-invasive optical methods. This is with the presupposition that the physiology of dengue patients during the febrile phase may show subtle patterns via optical spectroscopy.

### Diagnostic mechanism in dengue detection via spectroscopy

It is likely that if non-invasive dengue detection is possible, skin and tissue morphology will play a key role. Several symptoms and signs prevalent in dengue have been studied using optical methods. The superposition of spectroscopic patterns within dengue patients would thus likely be peculiar compared to non-dengue patients.

For instance, the most recent linkage of non-invasive spectroscopic methods with dengue victims was only reported during the plasma leakage (generally during defervescence) phase in 2014 by Soller et. al [19] with scarcity in subsequent studies to affirm or negate the findings. There are no studies as such in the febrile phase. However, manifestations of plasma leakage prior to defervescence, is often not easily detected and was only demonstrated using ultrasound during the febrile phase in severe cases [20].

Also, changes in serological composition and capillary leakage during the defervescence period may be detectable [19]. Also, several spectroscopic methods have reported prognostic value albeit not attempted to be directly linked to dengue patients. In a comprehensive review report, Raman spectroscopy on the blood has been reportedly capable of distinguishing oxy- or deoxyhemoglobin (Hb), leucocytes, platelets (possibly illuminating thrombocytopenic patients’ spectral particularity) and plasma as well [21]. Since most of the near infrared (NIR) light is mainly absorbed by water in the body [22], and capillary leakage consisting mostly of water, this feature of the symptom may return a distinct profile for a dengue patient.

**Fig 1:**
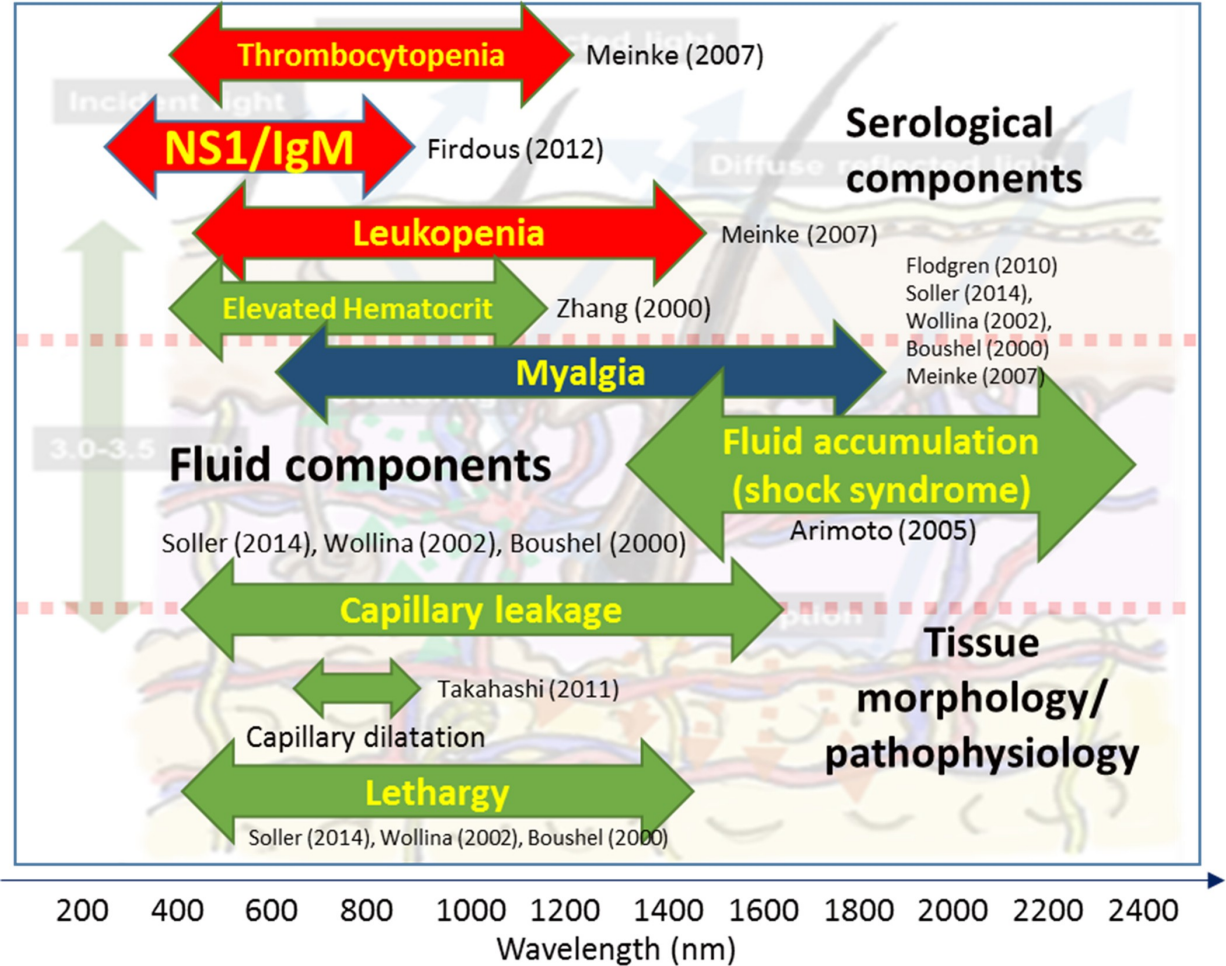
Spectroscopic wavelengths related/semi-related to dengue pathophysiology.

There also has been reports on differentiated transmission spectra of dengue-infected human serum without any reagent-based treatment, which was attributed to the absorption of dead blood cells [23], though debatable. The human tissue morphology and membrane topology in dengue patients has reportedly shown observable differences via in-vitro and histological studies [24, 25]. Moving to the visually apparent symptoms, the skin structure and manifestations of dengue have reportedly made significant correlation, especially among children. This is evident from hemorrhagic tendencies and cutaneous manifestations among patients during the febrile phase, including pruritus, petechial eruption, macular and papular skin rashes [26–28]. The dermatological features occurring from such manifestations are partly due to the capillary dilatation in patients associated with dengue fever [29]. In literature, the link on dermatological manifestations of dengue patients with optical skin spectroscopy has been virtually absent. Since these features may have its precursors undetectable to the human eye, it would be an optical method lending to its sensitivity for any combination of patterns, making an in-vivo investigation feasible. Therefore, it can be reasonably hypothesized that in its febrile and critical phase, dengue-stricken patients may elicit quasi-unique in-vivo spectroscopic characteristics resulting from the superposition of the various pathophysiological conditions which may be isolated to further assist diagnostics. The graphical summary for this section is shown in **Fig 1**, detailing wavelengths utilized in spectroscopic research which are related or semi-related to the pathophysiology of dengue. Some of the reports are based on ex-vivo tests, while the most are in-vivo. For this we put this hypothesis to test.

## II. Materials and methods

The following focuses on several tools including the technical and managerial perspectives in the data collection and analysis.

### Spectroscopy setup

**Fig 2:**
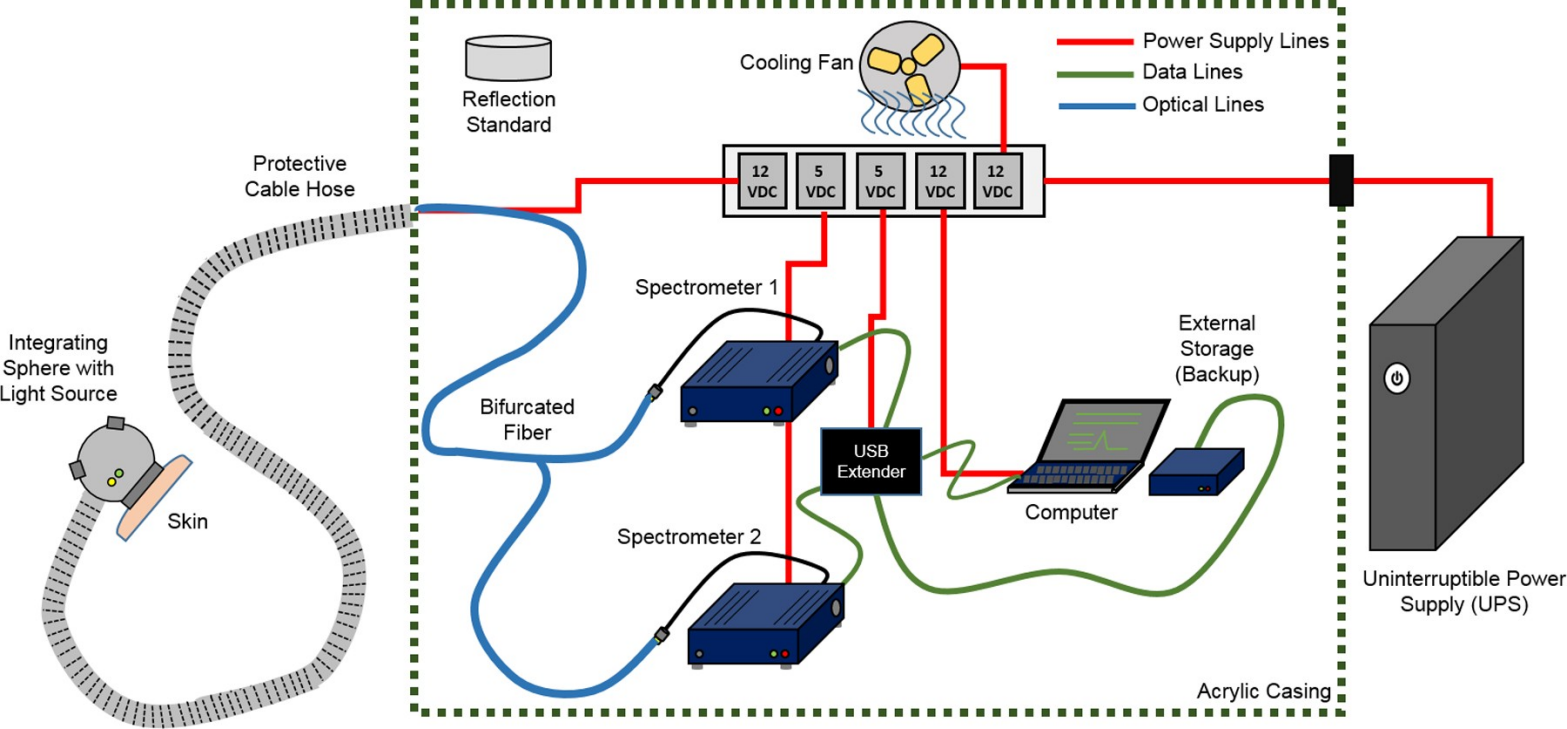
Spectroscopy setup.

**Fig 2** shows the schematic of the setup. We employ the use of diffuse reflection spectroscopy (DRS) in the UV-VIS-NIR wavelengths (200–2500 nm) which has reportedly higher sensitivity for in-vivo measurements of human skin profile. The spectroscopy data collection is centred on two primary spectrometers, covering between 200–1100 nm (Avaspec-3648, Avantes) and 900–2500 nm (NIRQuest 256–2.5, Ocean Optics). The Avaspec-3648 is simultaneously USB-powered and connected to a laptop computer via a separately DC powered USB port, running on the freely available software for operating its features (Avasoft 8, Avantes). The NIRQuest 256–2.5, operated using its own software (OceanView, Ocean Optics) is also USB-connected, albeit with an external 5V and 3A DC power source. Biological use of these spectrometer types have also been covered recently [30, 31].

Both spectrometers are hex-nut SMA-connected via a Y-shaped bifurcated fiber (Ocean Optics) where the labels correspond to the two diverging ends for wavelength range for each extension of the fiber. The common part of the fiber is connected to an integrating sphere with an in-built light source (AvaSphere-50-LS-HAL-12V, Avantes) is used as a probe to capture diffuse reflection spectra from the patients. The light source covers between 360-2500nm as per the specs furnished. However, it covers a wider UV region as well as tested with the Avaspec spectrometer, extending its use between 173-2500nm. The sample port diameter of the integrating sphere is 10 mm, an internal diameter of 50 mm and a stability drift of 0.1% per hour. Only two cables extend from the setup, where the first is the 12V DC, 1A power source for the built-in tungsten-halogen light source within the integrating sphere. The second cable is the common end of the bifurcated fiber. Both extensions are covered with a flexible plastic hose for external snag protection.

This setup is integrated into an acrylic housing for mobility in the site of concern, connected to an uninterruptible power supply (Cyber Power UPS, UT600E) allowing the setup to be ambulatory for recruiting patients in other areas. The setup is stored in a secure location after the data collecting session.

### Ethics statement and recruitment criteria

This study was approved by the University of Malaya Medical Centre (UMMC, Kuala Lumpur, Malaysia) Medical Ethics Committee (reference number 201511–1904). Written consent forms were obtained from each recruited patient. For patients under the age 18, written consent was obtained from the parents or corresponding guardian.

Patients above the age 12, undergoing triaging process from the Secondary Triage Area in Emergency Department (ED) of University of Malaya Medical Centre (UMMC), Kuala Lumpur were recruited in this study. <2% of the patients were recruited from other venues around the ED to maximize data intake. Patients who presented with a documented fever of > = 37.5°C and symptoms to suggest dengue, such as myalgia and arthralgia were screened. The patients are directed to the data collection site placed adjacent to the triage area, where written consent is obtained from either patients or guardians. Patients which require immediate resuscitation upon having vital signs read are excluded in this study. The sample size determination, in our case, is irrelevant due to the pilot nature of our undertaking.

### Data collection & management

Recruited patients were seated and rested beside the setup. After turning on the light source, the integrating sphere probe, extending from the setup was used to measure the full diffuse reflection spectrum using a reflection standard (WS-1, Ocean Optics) to establish the reference level, followed by dark-reference measurement. This was performed for every session with a patient, where both Avaspec and Ocean Optics software caters with a single click for both references in the reflection mode. The reflection mode in Avasoft (corresponding to the Avaspec-3648) was set to 4.18 ms integration time, while in the OceanView software (corresponding to the NIRQuest 256–2.5) was set to 100 ms integration time, while the boxcar width is set to 10. Note that the disparity of parameters is of no concern to the integrity of the data collection since these values are maintained throughout the data collection period.

The probe was then further placed on the patients’ right forearm. This area was chosen as it is the most accessible and uniform skin area. The reflection spectra is then obtained by clicking on “**Save graph to files**” on the OceanView software, and “**Export >> Excel (New File)**” from Avasoft. This is performed three times. The files generated are in the form of Microsoft Excel files for Avasoft, and .txt files from OceanView. These files are then saved in a folder generated from a profile created from a custom-built .NET application via Visual Basic or VS2013.

Every patient is assigned with a patient ID number. Their demographical, clinical, laboratory and spectroscopy data were recorded under this number. Other key parameters are automatically saved such as the timestamp, used for tallying other upcoming details on the patient during the time frame.

The clinical data evaluation to confirm the patients’ diagnosis was later performed by physicians from University Malaya Medical Centre (UMMC) based on the clinical information available in the clinical notes, full blood count results and NS1 antigen and dengue serology where available. The physicians were not aware of the spectroscopy readings of the patients they evaluated. Patients were categorised into three groups—confirmed dengue, probable dengue, and non-dengue (control).

### Clinical discourse & data category

Confirmed dengue are those with 1) laboratory and clinical features consistent with dengue fever and have a positive NS1Ag and 2) laboratory and clinical features consistent with dengue fever and have evidence of IgM/IgG seroconversion. Probable dengue cases are those with laboratory and clinical features consistent with dengue fever but have a negative NS1Ag test or do not have serological evidence of seroconversion.

To elaborate, a patient is classified as confirmed dengue if a patient returned positive for NS1 with clinical presentation, including an onset of fever within less than 6 days, combined with signs of acute dengue infection, with signs of IgM/IgG seroconversion. A patient with positive readings of IgM will also be considered confirmed dengue if the fever onset is more than 5 days, combined with serological consistencies such as low white blood cell and platelet count (Organization et al., 2009).

Patients with IgM-seropositivity before day 5 of the fever onset are considered probable dengue, if presented with clinical consistency with dengue.

Patients with IgG-seropositivity will be approached with rigor, where follow-ups to the data available for the patients will be scrutinized. This includes looking up into follow-ups on the patient.

Those who do not fulfil the above criteria are categorized as control, after arriving at a non-dengue diagnosis.

## III. Results & discussion

### Demographics

The data collection commenced from 27^th^ April 2017 to 16^th^ January 2018. A total of 257 patients were recruited. 17 patients were excluded as the spectroscopic data was faulty due to calibration issues. Data from 240 subjects were analyzed.

Out of the recruits, 216 were Malaysians, with 114 (47.5%) Malays, 39 (16.3%) Chinese and 59 (24.6%) Indians. The remainder of the subjects include other ethnicities such as 3 (1.3%) East Malaysians and 2 (0.8%) Punjabis. 23 (9.6%) citizens were from countries other than Malaysia, including 7 (2.9%) Bangladeshis, 6 (2.5%) Indonesians, and 10 (4.1%) others. The Malay population was accurately represented in this case as per the Malaysia demographics, while the Chinese were slightly underrepresented and the Indians slightly overrepresented. There were 124 males (51.7%) from the primary dataset, versus 114 females (48.3%). In terms of age, the range was between 14–90 years old, with a median of 32 years old. A slight majority of the recruits were deemed dengue suspects during the triaging process, at 122 patients (50.8%). From the population prior to examination of clinical notes, 20 patients (8.3%) were initially identified as positive for NS1, 9 are positive for IgM (3.75%), and 9 positive for IgG (3.8%). On the other hand, there are 25 NS1-negative patients (10.4%), 16 IgM-negative (6.7%) and 9 IgG-negative (3.8%).

### Clinical confirmation and classification

240 patients fulfilled the inclusion and exclusion criteria and had spectroscopy data.

We were able to classify 230 patients into confirmed dengue/probable dengue and control cases. 5 subjects left the hospital before a complete clinical assessment can be performed. Three had insufficient notes to draw a diagnosis. Two remaining patients are possibly dengue patients, but there was not enough data to confirm this. These 2 patients were excluded from dengue analytics but kept for analytics on ethnicity and gender.

Of the 230 remaining patients, 28 were confirmed to have dengue, 8 had probable dengue and the others were non-dengue/control. The diagnosis of the non-dengue patients included upper respiratory tract infections (URTI), acute gastroenteritis (AGE), other acute undifferentiated febrile illness, urosespsis, both community and hospital acquired pneumonia, cellulitis, and others.

Refer to **S1 Clinical data**. **Table 1** summarizes the clinical details of patients with confirmed and probable dengue.

**Table 1:** Clinical evaluation of dengue patients.

ID	Fever day	NS1	IgM	IgG	Final Diagnosis	2009 (DWWS, DWOS, SD)	Comments
**P0008**	4	Not Detected	Not Detected	Detected	Confirmed	DWWS	NS1 positive at day 4 from Private GP
**P0011**	6	Detected	n/a	n/a	Confirmed	DWWS	NS1 and IgM positive at GP at D6
**P0016**	5	Detected	n/a	n/a	Confirmed	DWWS	NS1 detected on D5, clinical consistency
**P0024**	7	Not Detected	Detected	n/a	Confirmed	SD	IgM detected on D7, clinical consistency
**P0032**	3	Detected	n/a	n/a	Confirmed	DWOS	NS1 positive on D3, without warning signs
**P0060**	3	Detected	n/a	n/a	Confirmed	DWOS	NS1 positive on D3, clinical consistency
**P0080**	12	n/a	n/a	n/a	Probable	DWOS	Rapid test positive in private GP, query NS1/IgM/IgG. Since taken on D12 most likely IgM/IgG positive therefore can only be said to be probable.
**P0100**	3	Detected	n/a	n/a	Confirmed	DWOS	NS1 positive on D3, clinical consistency
**P0103**	6	Detected	n/a	n/a	Confirmed	DWWS	NS1 positive on D6, measurement done in recovery phase
**P0104**	2	Detected	n/a	n/a	Confirmed	DWOS	NS1 also positive from GP & minimal PV bleeding
**P0108**	5	Not Detected	Detected	n/a	Confirmed	DWWS	IgM positive at D7
**P0109**	6	Not Detected	Detected	n/a	Confirmed	DWWS	IgM positive at D7
**P0117**	4	Detected	n/a	n/a	Confirmed	DWOS	NS1 positive at D6
**P0120**	3	Detected	n/a	n/a	Confirmed	DWOS	NS1 positive at D3
**P0122**	3	Not Detected	Detected	n/a	Probable	DWWS	IgM positive at D3, probable dengue
**P0125**	4	Not Detected	Detected	n/a	Probable	DWWS	IgM positive at D4, probable dengue
**P0133**	3	Detected	n/a	n/a	Confirmed	DWOS	NS1 positive at D3 but FBC normal
**P0135**	2	Detected	n/a	n/a	Confirmed	DWWS	NS1 positive at D2 with HLH
**P0149**	3	Detected	n/a	n/a	Confirmed	DWWS	Clinically suggestive: Low WBC and platelets, went to Private Hospital, NS1 positive
**P0167**	5	Not Detected	Not Detected	Detected	Probable	DWWS	Clinical consistency with dengue: fever at D5, low WBC and Platelet
**P0180**	3	Detected	n/a	n/a	Confirmed	DWWS	NS1 positive day D3
**P0182**	6	Not Detected	Detected	n/a	Confirmed	DWOS	IgM positive day D6
**P0185**	5	Detected	n/a	n/a	Confirmed	DWWS	NS1 positive at D5, clinical consistency
**P0197**	3	Detected	n/a	n/a	Confirmed	DWWS	NS1 positive at D3
**P0207**	5	Detected	n/a	n/a	Confirmed	DWOS	NS1 positive at D5
**P0211**	4	Not Detected	Detected	n/a	Confirmed	DWWS	NS1 positive at day 3 from Private GP
**P0213**	5	Detected	n/a	n/a	Confirmed	DWWS	NS1 positive on D5, Measurements on D7
**P0223**	6	Detected	n/a	n/a	Confirmed	DWWS	NS1 positive day D6
**P0224**	8	Not Detected	Not Detected	Not Detected	Probable	DWOS	Verified from Clinical Notes: Had FBC in GP showing reducing trend in Platelet and WBC
**P0225**	4	Not Detected	Detected	n/a	Probable	DWWS	IgM positive day D4
**P0226**	4	Not Detected	Detected	n/a	Confirmed	DWWS	NS1 positive at day 3 from Private GP
**P0229**	4	Detected	n/a	n/a	Confirmed	DWWS	NS1 positive at D4
**P0231**	8	n/a	n/a	n/a	Probable	DWWS	Towards recovery, fever subsided during recruiting
**P0232**	4	Detected	n/a	n/a	Confirmed	DWWS	NS1 detected at GP, petechia, showing reducing WBC and platelet trends
**P0233**	5	Not Detected	Not Detected	Detected	Probable	DWOS	Clinical consistency with dengue
**P0234**	6	Detected	n/a	n/a	Confirmed	SD	NS1 positive D6, compensated shock

### Raw data analysis: Decomposing confounding factors

**Fig 3:**
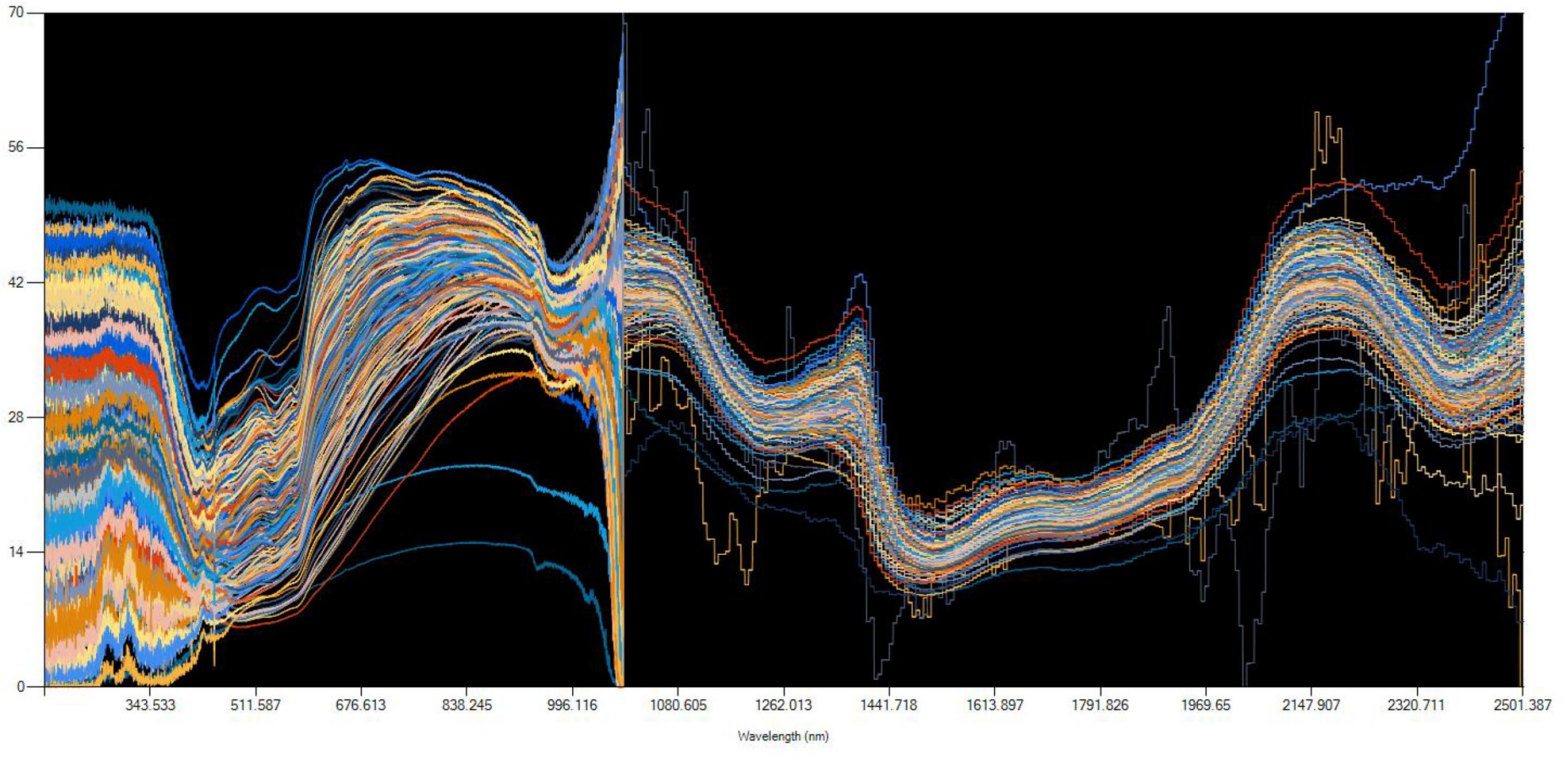
Overall view of the raw data.

**Fig 4:**
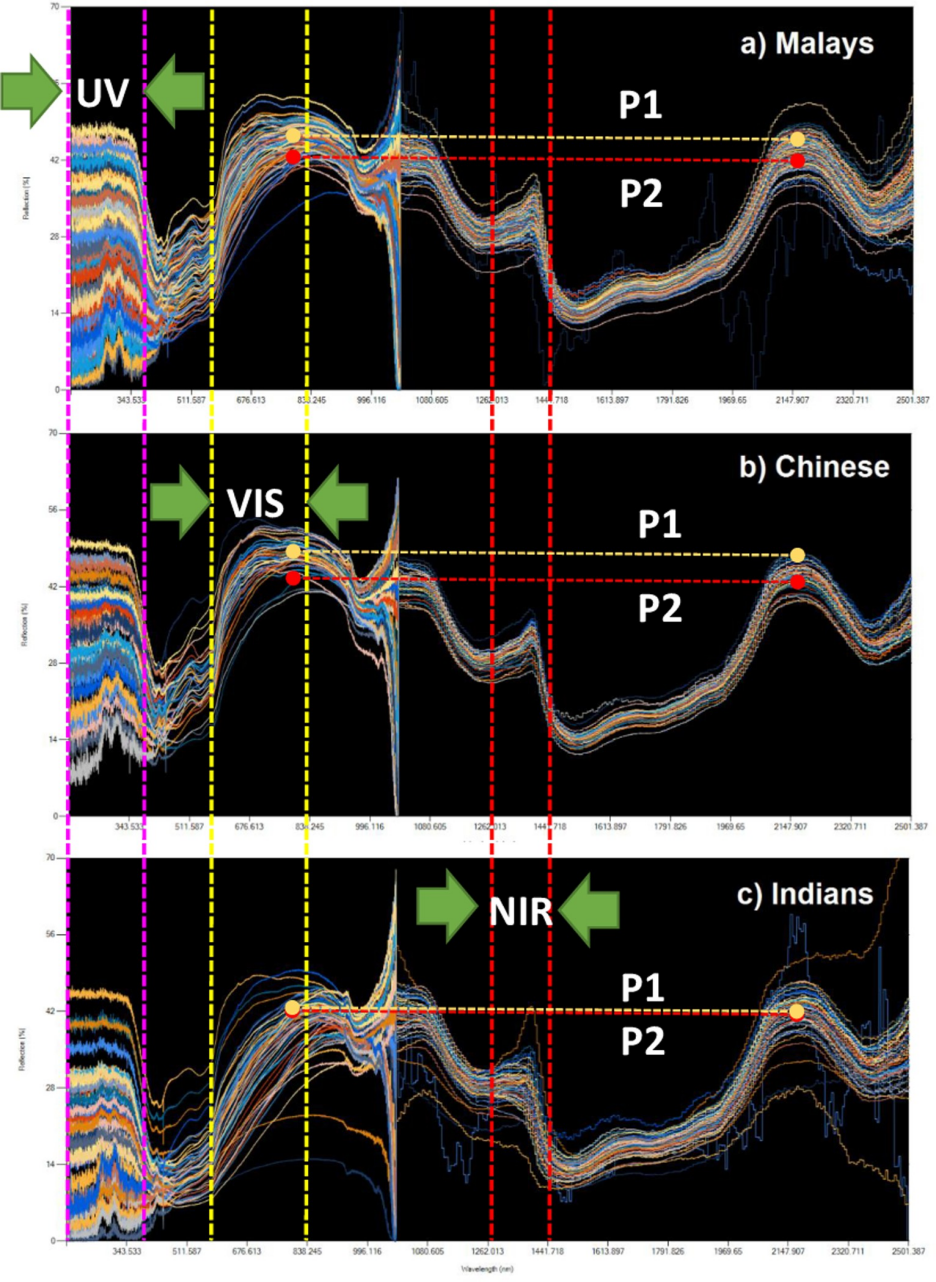
Decomposition of spectra between Malaysian Malays, Chinese and Indians.

In this section, apparent features of the data will be discussed to evaluate several salient issues and its relevance towards determining confounding factors in isolating dengue and non-dengue patients. **Fig 3** (rendered using the Chart control in Microsoft Visual Basic 2013) shows the raw data plots of the spectroscopic data of the population as a whole, depicting diffuse reflectance readings versus wavelengths between 173–2500 nm (**Refer to S1 Dataset**):

From **Fig 3**, several issues need to be clarified. In the NIR region (700–2500 nm), especially in 1100nm area as shown in **Fig 3**, the truncated appearance of the series is due to the concatenation of the spectroscopy data from two different spectrometers, with different settings due to the specifications. Therefore, it should be noted that consequent analysis from the dataset will reflect only the regions which has an acceptable level of consistency. This excludes the region from 1000.168–1069.836 nm (highlighted by the red dashed line limits) for readings taken from Avaspec-3645. This is due to its dissonance from the readings of its generally more consistent counterpart (NIRQuest spectrometer). This truncation is compensated by the readings from the NIRQuest spectrometer starting from 982 nm. Another potential concern is due to the relatively higher readings of the reflectance in the NIR region (700–2500 nm) compared to past literature, where reflectance readings are usually <10% due to water content in the skin [32–34], which is the main absorption agent of the radiation in the NIR. This is deliberately configured from the spectrometer settings for smoother graph outputs, a result from a relatively higher integration time of 100 ms. Since we were able to maintain consistency of the output readings, this configuration was standardized throughout the measurements during the data collection period. **Fig 4** in the following shows the three major ethnicities in Malaysia broken down to ethnic Malays, Chinese and Indians.

Based on the raw appearance of the data, different ethnicities exhibit noticeable difference especially in the visible region, as shown in the VIS section (390–700 nm) in **Fig 4**. This is intuitively the case as skin tone differences are usually discernible by the majority of people from a normal visual cue, as Indians generally have a darker skin tone than Malays, and the Chinese generally have a fairer complexion, as shown in **Fig 4**. In quantitative terms, the skin types are measured in terms of Fitzpatrick skin phototypes (SPTs), where Asians usually have types III, IV and V skin, where these classifications correspond to higher to lower reflectance percentages [35].

Another apparent pattern observed are the peaks *P1* and *P2*, which occurs between 370–920 nm and 2080–2250 nm respectively. Also, Indians apparently have a lower difference in the mean levels of the two peaks defined by *P1* and *P2* baselines as depicted in **Fig 4C**. On the same note, Malays have slightly lower peak differential compared to the Chinese ethnic. As shown in **Fig 6C**, in the **UV region** (150-400nm), Indians usually return a lower reading in the reflectance percentages due to the abundant absorption from melanin in the skin as reported in recent works as well [36], comparably similar in previous studies with Malaysian subjects [35]. In the VIS region, Indians also show a collectively lower reflectance reading which is due to the darker pigmentation in the skin. Although invisible to the naked eye, the highlighted NIR region also shows a negative slope for the Indians collectively. However, Chinese and Malays reflectance levels’ difference are not very significant although still noticeable, due to the blur between the SPT more prevalent among both ethnicities.

Elaborate multivariate analysis on the patterns is discussed in **S1 File** as a crucial aspect in determining normalization factors.

### Raw graph analysis for dengue-positive patients

**Fig 5:**
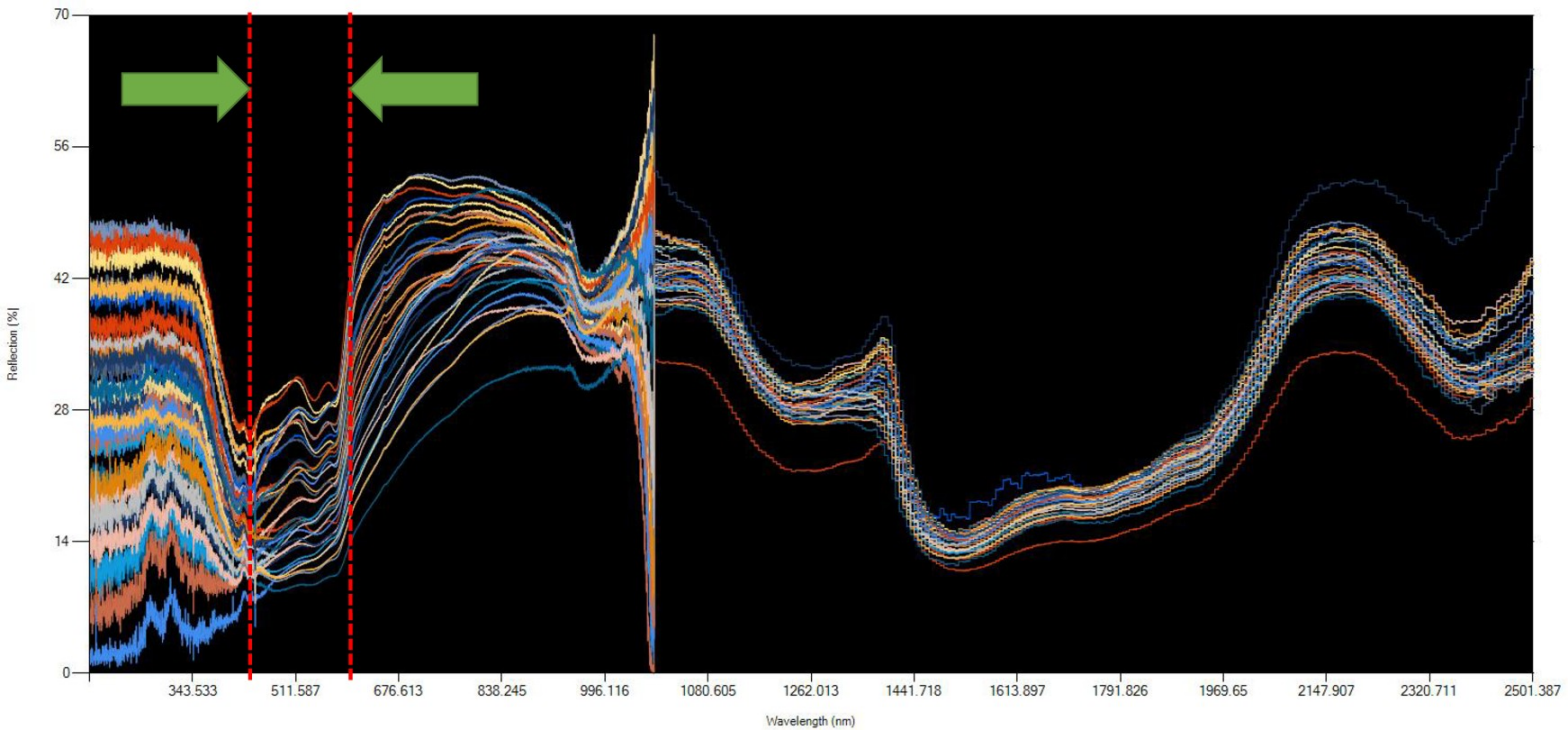
Raw spectroscopy pattern of confirmed and probable dengue patients.

**Fig 5** shows the spectra for confirmed and probable dengue patients.

From **Fig 5**, if any visual cues exist, the only apparent prevalence from visual inspection of the pattern is only in the VIS region between 457–585 nm. The pattern exhibits a double peak and trough in this region due to the complex tapestry of melanin, pigmentation and blood flow within the dermal substructures. However, this pattern is also prevalent in a sizable population from the general dataset. Therefore other cues from these patterns have to be analyzed more closely. For these features to be analyzed efficiently, there are many confounding factors which need to be considered. In previous studies, melanin content present in darker patients has shown to be one of the various confounding factors in skin spectroscopy [37].

Moving forward from this analysis, it is evident that the spectra exhibits a classic problem of closely-related species, or in our case, patterns. This calls for the use of multivariate methods, as discussed below.

### Discriminant analysis on dengue patients versus non-dengue patients: Classification accuracy

Based on feature-extracted data and principal component analysis (PCA) patterns (See **S1 File** on feature extracted data and PCA) of the whole data population, it is evident that confirmed and probable dengue patients exhibited a common structure in contrast to non-dengue, or control patients as well. In this section, we divide the analysis into three types, where a treatment of the multivariate techniques will be applied to three types of datasets. The first is the raw and untreated spectroscopy data, second only feature-extracted data, and third covers the combination of both of the aforementioned types to maximize the potential clustering appeal. See **S1 Dataset** for further info.

**Fig 6:**
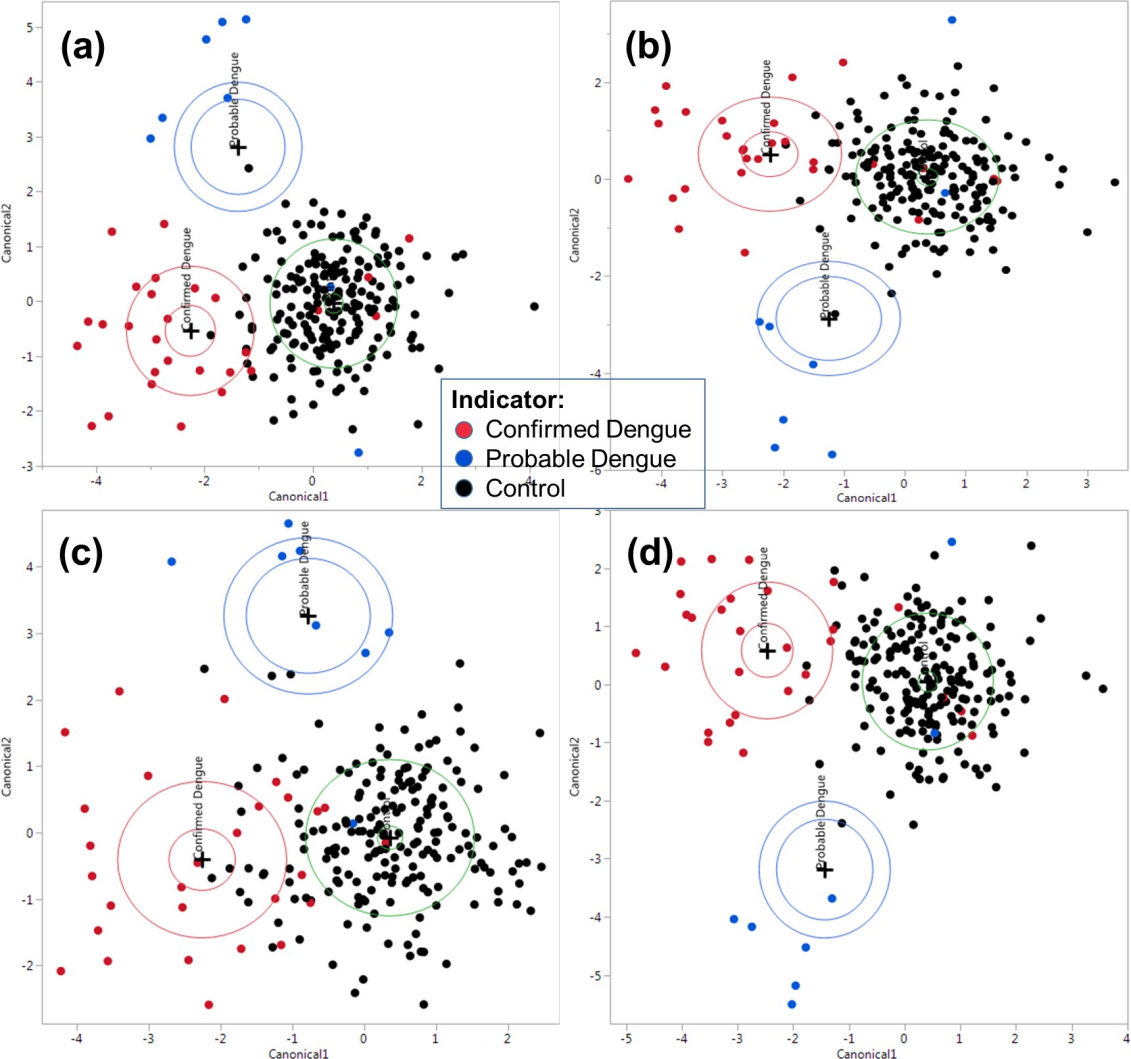
Wide Discriminant Analysis canonical plots on: (a) Raw-data, (b) Excluded noise data, (c) all feature-extracted parameters, and (d) combined Raw-Feature data. Two groups represent Confirmed and Probable dengue in contrast to the Control population.

As an attempt to advance the discourse on separating the dengue patients from the control, the use of discriminant analysis (DA) is used, as a conclusive tool in this section. Similar to the previous section, subsequent references to DA are all directly or indirectly computed using SAS JMP 12.2.0 (64 bit). To demonstrate the utility of DA in subsequent analyses, **Fig 6** in the following shows the canonical plots via Discriminant analysis of three data types.

Starting with **Fig 6A**, as with all the Discriminant plots in this section, a Wide Linear method is used, which optimizes the number of dimensions, computed from the principal components of the data. The data encompasses the complete range of raw spectroscopy values, which include the noise region (1000.168–1069.836 nm) as discussed in **Fig 4**. In order to analyze if the noise region influenced the canonical scores, we then exclude these regions. This comparison is performed to analytically determine whether a systematic selection of covariates into the covariance matrix is preferred over a Wide Linear method. The former would be preferable, provided the accuracy of the prediction is reasonable. In this case, the accuracy of the prediction is 90.87% with 10 control cases misclassified as confirmed dengue.

In **Fig 6B**, the raw data excludes the mentioned noise region for comparison in terms of the prediction accuracy. It can be demonstrated that the effect of the region is negligible for the modeling. This is due to the high variances generated by the covariates in the noise region renders it redundant for the classification. To quantify the quality of the scores, the percentage of misclassification of the samples returns exactly similar values.

In **Fig 6C**, the feature-extracted data, spanning 67 parameters, computed heuristically (See **S1 File** on feature extraction) shows a rudimentary presentation of the discriminant features as seen in **Fig 6A** and **6B**. Although the accuracy of the prediction is lesser than the predecessors at 80.87%, the minimal number of parameters, in contrast to ~3000 parameters in the previous analysis is worth noting.

**Fig 6D** shows the canonical plots for cascaded raw and feature-extracted data, with an improved classification accuracy at 93.48%, demonstrating utility in cascading different data types into the analysis. In the biplot, however, this is not too apparent as the DA classification algorithm operates in tightly defined boundaries.

The following section elaborates more on the demonstration of diagnostics, which represents the data in a binary outcome.

### Sensitivity and specificity: Prediction algorithms

As observed in **Fig 6**, the distribution of the canonical scores in the biplots demonstrated the spatiality of the patients in 2-dimensional space for three categories, namely confirmed and probable dengue versus control cases. This allows a probabilistic framework and manages dengue risk expectation on a continuum if were to be employed for screening purposes. However, for evaluation of diagnostic capability, standard measurements of sensitivity and specificity were used for the confirmed dengue and control cases only. This demonstrates the prediction algorithms from the statistical models computed from the discriminant analysis.

Probable cases, as a result, are excluded as a classification for a dichotomous case presentation using discriminant analysis (DA) by a statistical software (SAS, JMP 12.2.0). In this case, probable dengue cases (n = 8) is not assigned any classification, but retained in the analysis, allowing observation of the projection of the data on the plot. In turn, these cases provide a perspective on how the prediction algorithms from the statistical modeling will categorize each case based on the existing data. In the DA method, we optimize the outcome by weighing between prediction accuracy and the type of data used, which spans between the (a) raw data, (b) feature-extracted covariates, (c) vitals and finally (d) normalized data. Normalization on spectrum data based on non-quantifiable features of human patients has been reported on ethnicities [38] reportedly resulted in higher precision. However, there has been a lack of studies using gender as a normalization factor. In our cases, these two types of normalization factors are considered due to its ease of record. Also, both characteristics can be predicted with accuracies >90% from raw and feature spectrum values alone. See **S1 File** on ethnicities.

Based on classification algorithms from the statistical software, the three major Malaysian ethnicities can be quantitatively classified using multivariate methods. The main importance in highlighting the key differences of the ethnicities, however, lies in the practical classification of people not of the three main groups (Malays, Chinese and Indians) for spectrum normalization purposes. This is due to insufficient data to form an average ethnicity-based normalization reference for other Malaysian nationals, for instance Punjabis, and of non-Malaysians such as Bangladeshi workers, and foreign students. Our multivariate scores, in turn, help in categorizing each patient closest to the mentioned ethnicities resulting in a more accurate classification. This classification is later incorporated in the dataset with the declaration of the imputation of the ethnic skin type category, denoted within the dataset.

By normalizing the spectrum based on the ethnicity, the patterns of the spectrum can be augmented. Based on analyzing ethnicity, we arrived to a relatively consistent structure within the multivariate space, as demonstrated using PCA and Discriminant Analysis methods, with 91.5% accuracy in classifying the three major Malaysian ethnicities. The three categories of normalization is based on major ethnic groups in Malaysia, namely Malays, Chinese and Indians. For other ethnicities or nationals, the ethnicity on which the normalization occurs upon is based on the location of the scores on the canonical plots by the respective sample, as shown in **Fig S3(b)**. The normalization is performed by a straightforward truncation of the raw data from the average value of the corresponding class spectrum. Another normalizing factor is gender, where 93.5% classification accuracy from raw and feature spectra can be achieved. See **S1 File** on gender analysis.

The only non-spectroscopic data considered in our case is the use of vitals, which include temperature, systolic/diastolic rates, SpO_2_ and finally pulse rate. These parameters are mandatory measurements taken at triage. This renders it available as additional covariates for the classification algorithms. Also, in our case, the use of vitals increased accuracy of prediction algorithms between 1–2% in most cases. Several samples have to be imputed but the effect is negligible. By imputing parameters from vitals, and additional six parameters are gained. Some patients have their vitals imputed as per the guide for missing data [39]. However, this only occurs in 15 samples, or 6% of the population, mostly in respiratory rates. See **S1 Dataset** for details of the imputation.

However, since there are five types of data landscapes (basic, feature, vitals, ethnicity and gender), spanning more than 8000 covariates, there are endless permutations of normalization. Therefore, a few parametric convolution techniques are non-exhaustively described in **Table 2**. An optimal combination is defined as having the highest possible prediction accuracy, especially for confirmed dengue cases, with the least number of data types. In cases where the parameters overwhelmingly outnumber the sample size (p>>n), the Wide method DA is used, where the analysis of covariance in the data is performed through its principal components. This means the use of Linear DA is not considered for concerns of accuracy of the representation of the data.

**Table 2:** Specificity and sensitivity of different normalization techniques.

Function ID	Function denotation	Formula for Normalized Data	% Misclassified	% Accuracy	TP	FP	TN	FN	Sensitivity	Specificity (%)	Confirmed Dengue	Control
									(%)			
a	F(basic)	n/a (Raw Data)	6.3	93.7	24	10	184	4	85.71	94.85	3	5
b	F(basic & feature)	n/a (Raw + Feature)	4.95	95.05	24	7	187	4	85.71	96.39	3	5
c	F(basic & feature & vitals)	n/a (Raw + Feature + Vitals)	5.4	94.6	24	8	186	4	85.71	95.88	3	5
d	F(ethnic)	X_eth_ = X_raw_−X_eth_	5.41	94.59	22	6	188	4	84.62	96.91	1	7
e	F(gender)	X_gender_ = X_raw_—X_gender_	6.3	93.7	21	7	187	4	84.00	96.39	2	6
f	F(eth/gender)	X_eth_ / X_gender_	5.4	94.6	24	8	186	6	80.00	95.88	3	5
g	F(normalized(X_eth*&+X_gender))	(X_eth_ * X_gender_) / (X_eth_ + X_gender_)	1.35	98.65	25	0	194	3	89.29	100.00	1	7
h	F(normalized(X_eth*&-X_gender))	(X_eth_ * X_gender_) / (X_eth_—X_gender_)	6.3	93.7	21	7	187	7	75.00	96.39	2	6
i	F(Sqrt(eth^2 + gender^2))	Sqrt(X_eth_^2 + X_gen_^2)	5.85	94.15	23	8	186	5	82.14	95.88	3	5
j	F(product(X_eth & X_gender))	X_eth_ * X_gender_	6.31	93.69	24	10	184	4	85.71	94.85	3	5

TP: True Positive, TN: True Negative, FP: False Positive. Sensitivity = (TP/(TP + FN))%, Specificity = (TN/TN+FP)%

**Fig 7:**
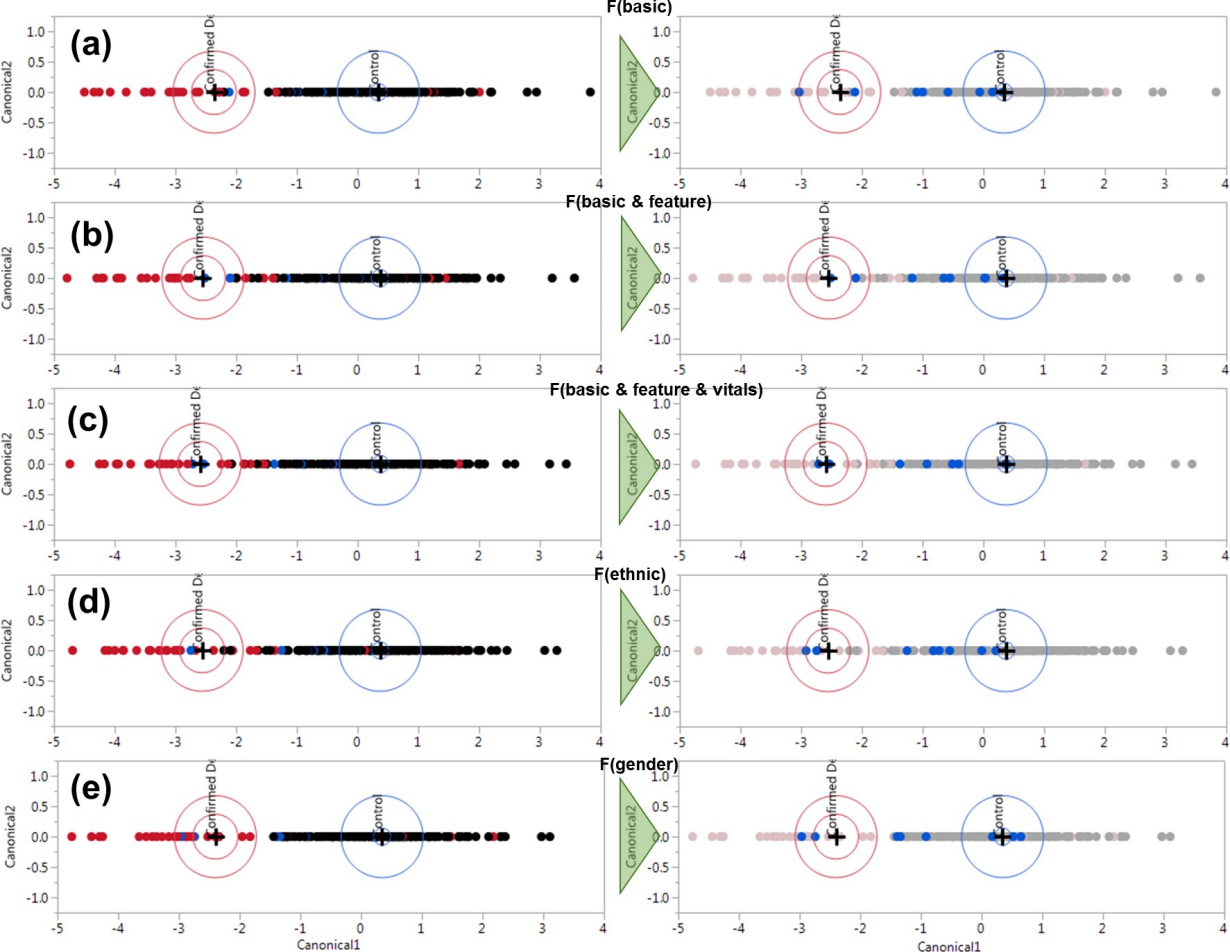
Canonical scores of discriminant analysis of different data types, with single-dimension plots of (a) basic (or raw) untreated data, (b) basic with added feature-extracted data, (c) basic, feature and vitals, (d) Ethnicity-based-only normalized data, and (e) Gender-normalized data.

**Fig 8:**
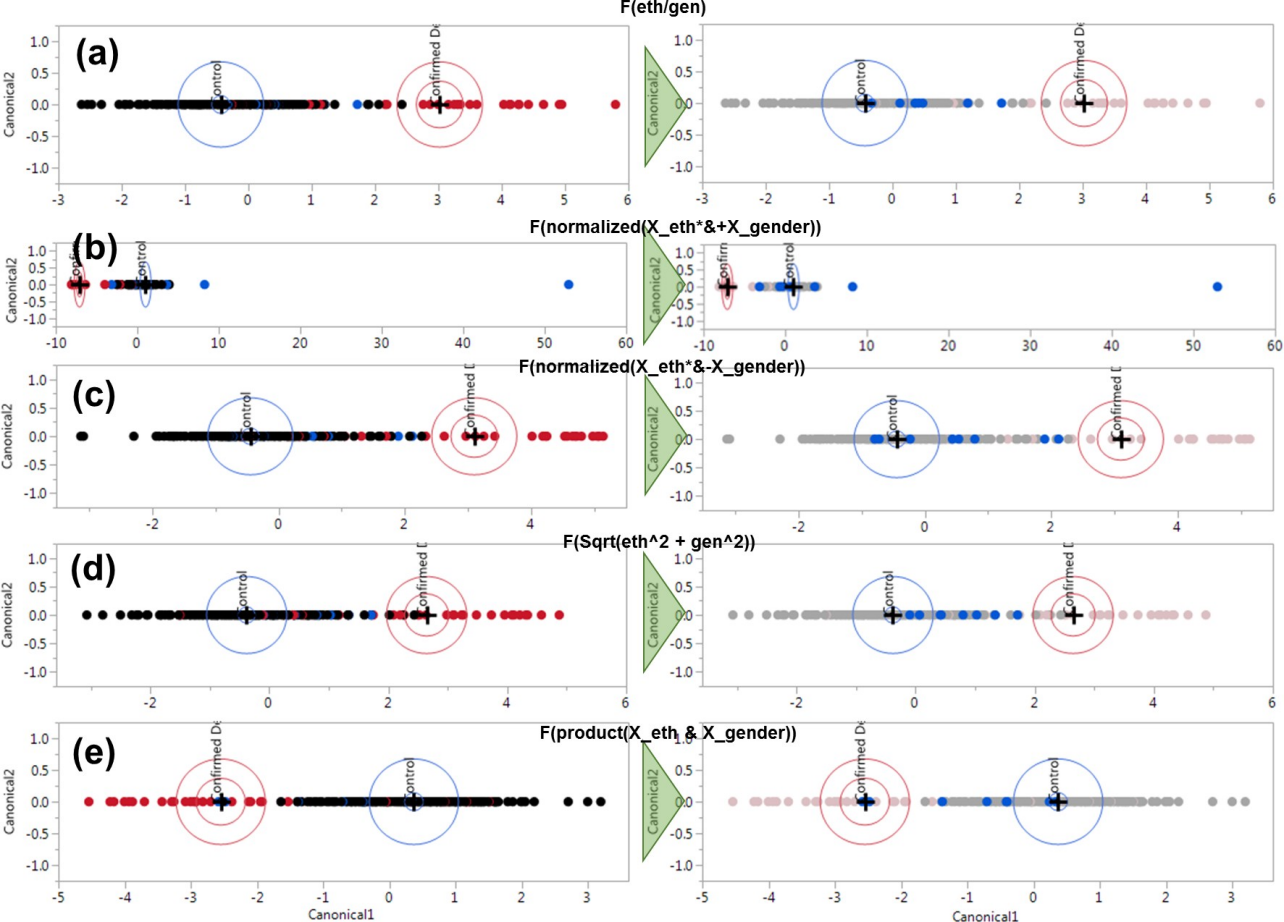
Canonical scores of discriminant analysis of different hybrid-normalized data types, with (a) F(eth/gender), (b)F(normalized(X_eth*&+X_gender)), (c) F(normalized(X_eth*&-X_gender)), (d) F(Sqrt(eth^2+gender^2)), (e) F(product(X_eth & X_gender)). Refer to **Table 2** for elaboration of the denotations.

To demonstrate a prediction pattern, the probable dengue cases were used as a test data. The distribution of the scores on the line plot, whether they fall into the confirmed dengue, or control regions can be observed. If we arbitrarily assume three out of eight probable cases are actually dengue, the most accurate model would end with the prediction score which falls in the confirmed category. The summarization of the discriminant analysis on different data types is shown in **Figs 7** and **8** respectively. See **S1 Dataset** for exhaustive details on the combinations of parameters used.

**Figs 7** and **8** shows the separation of confirmed and control cases on the left, where red dots represent confirmed dengue patients and black for control patients. The corresponding right-side figures correspond to the distribution of the probable cases, where the original confirmed and control cases are obscured to enhance the appearance of the probable cases. The probable cases (highlighted as blue dots) are unclassified in the modelling for observation purposes. It is to be noted that the measurements of sensitivity and specificity correspond to the diagnoses of each cases, rather than directly linking to the seropositivity results such as NS1, IgM and IgG.

**Fig 7A–7E** shows classification accuracies ranging between ~93–95%. Sensitivity and specificities of these methods, in turn, return ~84–85% and 94–96%. Out of 8 probable dengue cases, 3 cases steadily registered on the confirmed region, apparently demonstrating a relatively stable model. The *F(ethnic)* function, shown in **Fig 7D** demonstrates a lower level of sensitivity at 84.62% compared to **Fig 7A–7C**. It employs the use of all the available covariates in, including the vitals and raw spectrum as well, which is non-optimal. In this case, ethnicity-normalized data proves to be not advantageous.

**Fig 8A–8E** shows convolution between the ethnicities and gender-normalized data resulted in higher levels of accuracy. Each of the figures corresponds to the formula elaborated in **Table 2** from row **f-j** respectively. For these different hybrid convolution functions, **Fig 8A** seems to be the least optimal model, with 80% sensitivity. In contrast to the latter, function (g) as per **Table 2** (denoted by F(normalized (X_eth*&+X_gender))) shows the best fit for the statistical model, demonstrating a 89.29% sensitivity and 100% specificity. Also, it employs the least number of data types, which is dependent on the normalized hybrid ethnic-gender computation only, excluding vitals, feature covariates, and raw data. However, it classified only one probable dengue patient into the confirmed dengue class. This also will be a matter to be elucidated in future research.

In any case, if the apparently best model is actually proven to be inaccurate in future, the alternative models which can be considered are function (b) as shown in Fig 7B, which is simply cascaded raw and feature-extracted data with accuracy of 95.05% and sensitivity of 85.71%, specificity of 96.39% and registering 3 confirmed dengue patients out of the probable dengue case pool.

In comparison to current methods, several diagnostic kits and their respective sensitivity and specificities, especially serological tests have yielded slightly higher points of accuracy. Nathanson et al summarized in 2010 a detailed review the ~90% sensitivity and 98% specificity of IgM-based assays. Rapid test kits were more varied, with 21–99% sensitivity and 77–98% specificity [40]. More recently, in comparison from diagnostic methods of similar bearing, a recent rapid detection test (RDT) which is a combination of an NS1 and IgM detection yields percentages of sensitivity of 88.65% and specificity of 98.75% [41].

The mechanisms responsible for peculiarities in the spectrum enabling the discrimination of dengue patients from the control population remains speculative and challenging to be extricated at the moment. This is due to the complex nature and turbidity of skin tissues comprising of dermis, epidermis, sweat glands, fat tissues and blood capillaries. We propose a topological or morphological tissue structure peculiarity of dengue-infected patients. Several reasons to this theory are elaborated as follows. One is due to the tissue oxygenation levels, attributed to capillary perfusion which may render during transitions toward defervescence in dengue patients [19], contributing to the structure of the reflectance spectrum. Another angle is the contribution of inflammation of dermal dentritic cells (DC), reportedly being heterogenous throughout the skin of dengue-infected patients [42]. With Raman and near-infrared spectroscopy (NIRS), skin with melanoma can be differentiated from healthy tissues due to the enlargement of cancer cells, occuring at molecular levels, and capable of detecting cell inflammation as well [43, 44]. In conjunction for dengue, it is arguable that the overall skin topology may have been affected due to the elevated presence of deposited dengue viral antigens in endothelial cells [25], though is unclear at the moment if the forearm bears the same conditions, and if this is measurable under our setup. From the viewpoint of hematology, we reserve upon the contribution of the blood components, especially dengue antigens in the blood capillaries. This is due to the use of visible spectroscopy has reportedly shown differentiation capability for detection IgM or IgG (although debatable) in the blood sera, with observable absorption occurring in the 400–1000 nm region in contrast to non-dengue samples [23]. In our case, however, this was not observed as shown in **Fig 5**, most likely due to the occlusion from the turbid skin structure. However, its subtle contributions to the spectra may be justified, in accordance to Beer-Lambert’s Law stating that the absorption of the light is proportional to concentration of the chromophores in the solution, in this case the blood components and plasma [45].

## Conclusion

In conclusion, we have demonstrated a first case of possible use of a point-of-care (POC) diffuse reflectance spectroscopy setup for in-vivo screening dengue patients. This is performed by analyzing 240 patients of different age, ethnicity and genders. The data is then clinically verified by cross-referencing with clinical notes by Infectious Diseases (ID) and Emergency Department (ED) physicians. Diagnostics on each patient is further divided into three classes, namely confirmed dengue, probable dengue and control cases, comprising of febrile patients, except for two patients who were afebrile.

The spectroscopy data is later treated under different normalization techniques and statistically examined using Principal Component Analysis (PCA) and Discriminant Analysis (DA) techniques. The DA methods yielded accuracies between 93–98%, with sensitivity and specificity percentages ranging between 75–89% and 94–100%. However, in our estimation, due to the unlikeliness of any diagnostics scheme achieving unity in accuracy readings, the most accurate model we produced is to be approached with caution, and open to reassessment and critique. Currently, the most promising model seems to be F(normalized(X_eth*&+X_gender)), as shown in **Fig 8B** and **Table 2** (denoted by function (g)) which has a sensitivity and specificity of 89.29% and 100%, though confirming only one out of eight probable dengue cases, with a heuristically-driven rule.

The detection scheme, if applied, requires less than 60 seconds to conclude using multivariate methods. This study, to the best of our knowledge, pioneers an attempt at bypassing invasive methods of dengue diagnostics, with immediate results. Future investigations on verifying these results are solicited and welcomed for further understanding on the issue.

## Supporting information

S1 Dataset All spectroscopic data of each patient are compiled in this file. (XLSX)

S1 Clinical data All diagnostics of the patients are provided in this file, including confirmed dengue, probable dengue and non-dengue control. (XLSX)

S1 File Features and normalization. The features and confounding factors in the spectroscopic data are discussed at length in this document. [35–37, 46–54].
(DOCX)
