# Supplementary material for: Precursors to non-invasive clinical dengue screening: Multivariate signature analysis of in-vivo diffuse skin reflectance spectroscopy on febrile patients in Malaysia
Source: PLoS One. 2020 Apr 1;15(4):e0228923. doi: 10.1371/journal.pone.0228923 (PMC7112162; doi:10.1371/journal.pone.0228923)
Supplement: S1 File — The features and confounding factors in the spectroscopic data are discussed at length in this document. [35–37, 46–54]. (DOCX) [file pone.0228923.s003.docx]

**Supporting Info 1: Features and Normalization**

This section elaborates on the technical aspects on treating the data for augmentation. Three main aspects will be discussed. The first covers on feature extraction, which is intrinsic within the spectrum. The second deals with the ethnicity analysis and its relevance for data normalization and feature extraction. The third section discusses the use of principal component analysis (PCA) for determining structure in the data for dengue patients. The fourth section discusses gender as a confounding factor – hence a normalization factor.

1. **The requirement of feature extraction: A case point**

Based on the scattering of the data, the spatiality of the laboratory-confirmed NS1- and IgM-positive patients does not exhibit any form of variance from the general population from a visual inspection. Therefore a technique on deriving apparent patterns is devised for further elucidation of the peculiarities of the patterns.

In order to formulate the intrinsic patterns of the data, whilst enabling this to be performed independent of other datasets, a feature extraction method is devised. This method allows for an internal normalization, which offsets the need for comparative normalization with the whole dataset, where a direct normalization is deemed impossible due to the fuzzy boundaries between the ethnicities due to their SPT classes as discussed in **Fig 2**. The features of the data are computed from peaks or troughs in the pattern, numerically compared with heuristically generated baselines in reference to the peaks based on intrinsic proximity. This allows for a more straightforward and more accurate representation of the data. On another note, comparison between other data tables on the raw values also present another confounding factor due to the non-uniformity in applying probe pressure (in this case, the integrating sphere) on the patients’ skin, as discussed by previous researchers on this topic [[1](#_ENREF_1)]. This is due to the blanching of the skin where the constituents of the chromophores in the cutaneous and subcutaneous components become significantly conflated [[2](#_ENREF_2), [3](#_ENREF_3)]. To compensate for this compression issue, this method is used. To the authors’ knowledge, this method is considered novel for this specific application, where the closest form of such this method has been reported on the use of feature extraction on digitized images [[4](#_ENREF_4)], which is a different data type. However, none was reported on skin spectroscopy patterns.

**Heuristics on defining features**

Basically, there are several feature types in the pattern which may be extracted. Based on our observation, there are five classes of features which can be observed. The first are apparent peaks in the data which are selected by accessing the scalar reflectance percentage values. Second, this is followed by the scalar values of the peaks formed as discussed from **Fig 3**. Third is the formation of the UV baseline (175-285 nm), which is often a confounding factor in many measurements due to melanin in the skin [[5](#_ENREF_5)]. The fourth are all apparent gradients and intersections of the pattern, which is formulated as an attempted method for compensating the effect of probe pressure on the skin. This is due to the instability of the spectroscopic data which shows linear fluctuations after the integrating sphere probe has come into contact to the skin [[1](#_ENREF_1)]. The slope is computed as follows:

$$m_{n}= \frac{{P_{\lambda_{2}}-P}_{\lambda_{1}}}{\lambda_{2}-\lambda_{1}}$$

With m_n_ denotes the n-th gradient, P _λ2_ and P _λ1_ are the reflection percentages corresponding to the selected wavelength of both Cartesian points in the column and both λ_2_ and λ_1_ are the corresponding wavelength range values. The linear intersection of the corresponding gradient, however, provides an amplification of the scalar features of the data and is a tool which reserves use in certain conditions, and enumerated as follows:

$$c_{n}=P_{\lambda_{2}}- \frac{{P_{\lambda_{2}}-P}_{\lambda_{1}}}{\lambda_{2}-\lambda_{1}}*\lambda_{2}$$

Or simplified as follows:

$$c_{n}=P_{\lambda_{2}}- m_{n}*\lambda_{2}$$

Where c_n_ denotes the y-intersection value. The fifth and final feature are several small-scaled cues which exhibits salience (<2% reflection from baselines) from the data which may be important in the classification, which is all in the VIS region. Table 2 elaborates on the selected features and the wavelengths.

**Table S1: Features and corresponding wavelengths**

| Major Features | Wavelengths (nm) | Note |
| --- | --- | --- |
| a) Scalar Values | | |
| P_1_ | 650.00-850.00 | Peak values in both VIS and NIR |
| P_2_ | 2100.00-2250.00 |  |
| Q_1_ | 515.92 | Values of the double/ troughs. If these peaks/troughs do not exist, scalar value is taken. |
| Q_2_ | 545.51 |  |
| Q_3_ | 566.73 |  |
| Q_4_ | 575.00 |  |
| Baseline | 175.00-285.00 | Average value along this band |
| b) Linear Gradient & Intersection Values | | |
| m_1_ , c_1_ | 485.23 - 505.25 | Slopes in VIS and NIR. Note that m_9_ is predominantly an important factor in isolation in ethnicity.  All in the form of a range. |
| m_2_, c_2_ | 520.25 - 531.89 |  |
| m_3_, c_3_ | 551.82 - 561.76 |  |
| m_4_, c_4_ | 566.73 - 571.70 |  |
| m_5_, c_5_ | 735.54 - 752.05 |  |
| m_6_, c_6_ | 767.89 - 784.34 |  |
| m_7_, c_7_ | 961.41 - 970.50 |  |
| m_8_, c_8_ | 976.76 - 989.25 |  |
| m_9_, c_9_ | 1332.79 - 1371.29 |  |
| m_10_, c_10_ | 1390.52 - 1435.32 |  |
| m_11_, c_11_ | 1435.32 - 1480.06 |  |
| m_12_, c_12_ | 1531.10 - 1588.44 |  |
| m_13_, c_13_ | 1690.20 - 1734.67 |  |
| m_14_, c_14_ | 1766.43 - 1861.67 |  |
| m_15_, c_15_ | 1956.94 - 2020.51 |  |
| m_16_, c_16_ | 2052.33 - 2084.16 |  |
| m_17_, c_17_ | 2243.77 - 2275.80 |  |
| m_18_, c_18_ | 2417.28 - 2481.94 |  |
| m_19_, c_19_ | 1093.63 - 1158.59 |  |
| m_20_, c_20_ | 995.65-1041.458 |  |
| m_21_, c_21_ | 242.09 - 275.89 | Minor gradients in UV region. All in the form of wavelength range. |
| m_22_, c_22_ | 275.89 - 295.31 |  |
| m_23_, c_23_ | 295.31 - 310.27 |  |
| m_24_, c_24_ | 310.27 - 327.25 |  |
| m_25_, c_25_ | 644.20-676.94 | Minor gradients in VIS and NIR. |
| m_26_, c_26_ | 594.83 - 611.32 |  |
| m_27_, c_27_ | 360.80 - 394.58 |  |
| m_28_, c_28_ | 841.76 - 870.45 |  |
| Minor Features | | |
| VIS_1_ | 409.41 | A minor peak relative to the surrounding baseline i.e minus the value at 409.1 nm or 409.7 nm |
| VIS_2_ | 446.05 | Values relative to a baseline which is defined around 445.04 nm |
| VIS_3_ | 426.235 |  |
| VIS_4_ | 652.394 | Relative to the baseline value at 649.12 nm |

**Fig S2** in following demonstrates a feature extraction from the slopes and minor features from the data, by manually introducing the gradients in the selected region in the graphs.


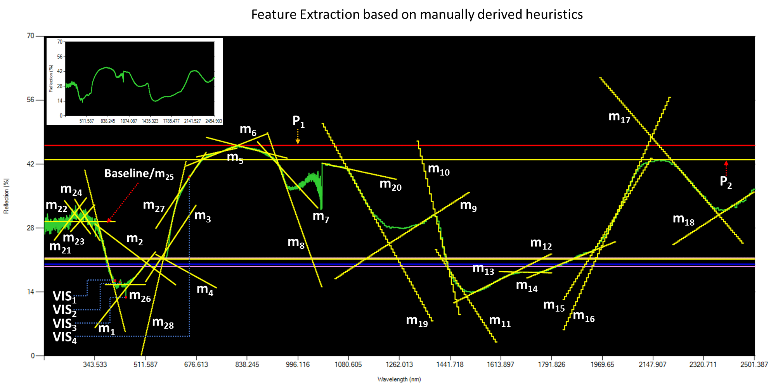


**Fig S2. Example of feature extraction decomposition. Inset shows the original form of the graph.**

There are several advantages of extracting features in this manner, which includes the ability to amplify the variance of each data. This can be demonstrated in two cases from the dataset where raw graph plots show virtually similar patterns which show radically different patterns by extrapolating the features. The following section demonstrates the use of multivariate analysis on the features.

1. **Ethnicity Analysis: Demonstrating viability of the feature extraction method**

To put the data consistency and feature extraction method to test, using a statistical software (SAS, JMP 12.2.0), **Fig S3** in the following demonstrates its utility in isolating several features of the data for a multivariate analysis. A Principal Component Analysis method was used by isolating m_1_ to m_12_, c_1_ to c_12_ and the Baseline parameters into the biplot. This achieves a dimensional reduction required to further classify the data.


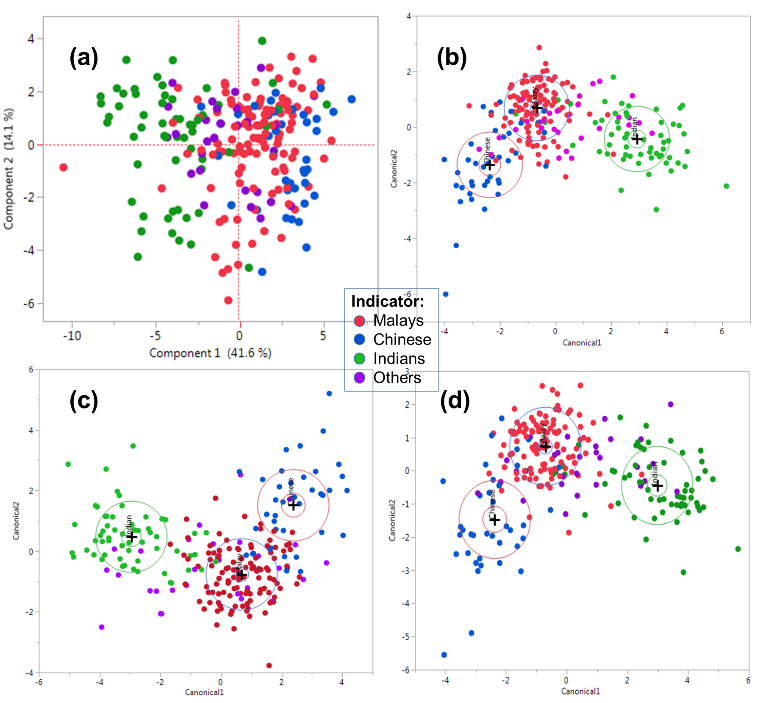


**Fig S3. Ethnicity profile represented by (a) PCA biplot of feature-extracted parameters, where the others show Discriminant Analysis biplots from (b) raw spectroscopic data, (c) Feature-extracted parameters, and (d) combined Raw-Feature parameters.**

From **Fig S3**, it can be noted that this technique shows the viability of the feature extraction method in classifying ethnicities to a reasonable extent. The red, blue, green and purple dots represent Malays, Chinese, Indians, and others respectively. The presence of other ethnicities in the otherwise discernible ranges shows anomalies only due to the fuzzy overlaps of skin phototypes of Asians, as reported in previous research [[6](#_ENREF_6)], where a minority of Indians can have SPT type III too [[7](#_ENREF_7)], and this would hold to be true in other classes as well. Predictably, the inclusion of the feature-extracted intersection values (c_1_-c_12_) allows optimizing cluster appearance for discriminating the ethnicity. This is intuitive due to the obvious appearance of skin tones to the naked eye. Also, the computation of c_1_-c_12_ values were intended to augment the raw spectrum data, including in the VIS region (390-700 nm). Also, the *Baseline* parameter which represents the **UV region** (150-400nm) is more pronounced in the Indian group, which allows more divergence of the latter from other groups, as supported in earlier literature [[6](#_ENREF_6), [7](#_ENREF_7)]. However, a detailed analysis on the scores on this analysis will be excluded for the sake of brevity.

**Figs S3(b-d)** deals with Discriminant Analysis techniques, which exhibits the three main groups reasonably. Similar to **S3(a)**, **Fig** **S3(b)**, which is limited to the raw spectroscopy data values, shows the divergence of Indian subjects from the other classes whereby ethnic Malays and Chinese tend to intersect, an attribute which is predictable based on the prevalence of Malaysians’ medium skin phototypes (III-IV) [[8](#_ENREF_8), [9](#_ENREF_9)]. **Fig S3(c)** shows the use of feature-extracted parameters which separates the classes reasonably well, although not as precise compared to **Fig S3(b)**. This is due to the features which were not discernible from the raw data, but would allow the optimum separation. However, the scores of the canonical plots from the analysis shows the viable use of the features, which reduce the range of dimensions significantly. However, a combined raw and feature-extracted data yields no significant difference in terms of the statistical prediction algorithms, as shown in **Fig** **S3(d)**. The summaries of the classification accuracy is shown in Table 3. These values are based on the classification obtained from the JMP statistical software after employment of the discriminant analysis. In our case for the latter, all parameters were included for the consideration, without selective parameters considered unlike a stepwise linear method.

**Table S2. Classification prediction scores**

| Data types | Number Misclassified | Percent Misclassified | Entropy RSquare | -2LogLikelihood |
| --- | --- | --- | --- | --- |
| Raw | 20 | 9.47867 | 0.6618 | 142.675 |
| Feature | 38 | 18.0095 | 0.61365 | 162.985 |
| Raw + Feature | 20 | 9.47867 | 0.69035 | 130.63 |

**Table S2** demonstrates the use of features for ethnicity analysis, however, does not increase the accuracy.

#### **Principal Component Analysis: Finding structure**

A principal component analysis (PCA) bi-plot is shown in **Fig S4**, which illustrates the three groups – namely confirmed and probable dengue cases, and other clinically non-dengue patients as control. Subsequent references to PCA are all directly or indirectly computed using SAS JMP 12.2.0 (64 bit).


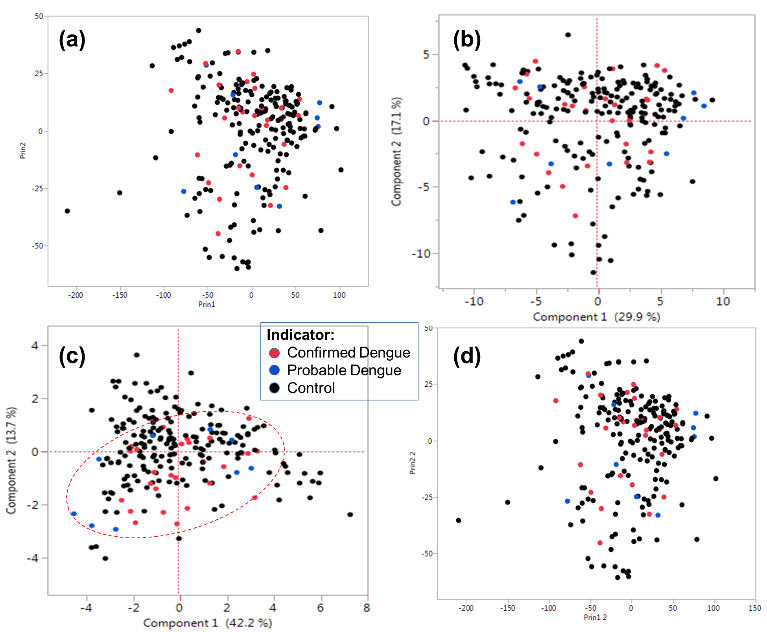


**Fig S4. PCA biplot of: (a) Raw-data, (b) All feature-extracted parameters, (c) selective feature-extracted parameters, and (d) combined Raw-Features. The red and blue dots represent confirmed and probable dengue patients, and the remainder black dots represent control patients.**

**Fig S4** shows the PCA biplot of several modes of datasets. **Fig S4(a)** shows the most variability along the first component (component 1), though no clustering tendencies appear from the biplot. **Fig S3(b)** further illuminates the PCA plots from the features of the data, which amount to 67 data points representing various parameters (compared to 2900+ raw data points) included in the analysis in contrast to the more voluminous raw data as shown in 8(a), while allowing the scattering of the variability at apparently higher ratio in Component 1 compared to Component 2.

To maximize convergence of the confirmed and probable dengue clusters, the selection of the parameters included for the PCA are methodically performed. By selective PCA, **Fig S3(c)** shows a tendency for the clustering appeal, and hypothetically allows us to project a map on the biplot as shown in the form of the red dotted ellipse. To achieve optimal measurement complexity [[10](#_ENREF_10)], the selection of the parameters for this plot is achieved by examining various combinations of the parameters. Parts of the feature which dominate the hemoglobin content accessible from the skin is around 410, 510 and 575 nm respectively, whereas the water band occupies 970 and 1450 nm [[11](#_ENREF_11)]. These wavelengths are part of the coverage between m_1_ to m_12_ (refer table 2), while excluding its intersection counterparts c_1_-c_12_. These values are independent of the actual value of the raw data due to its gradient nature. Furthermore, the exclusion of the noise band, occurring around 990-1100 nm as shown in **Fig 2** from the feature extracted profiles allows a higher tendency for the convergence. Also, it seems from the structure of the data, the exclusion of an ethnically-dependent region such as the UV, represented by the *Baseline* parameter (refer to **Table S1**) allows for the apparent convergence of the confirmed and probable dengue cases. However, it can also be concluded that even with informed decisions of inclusion or exclusion of the data points, a satisfactory structure which allows separation of dengue and control patients is not possible with PCA.

However, almost identically to **Fig S4(a)**, a combination of raw-feature data PCA plot does not enhance nor exhibit any useful structure on the data, as shown in **Fig S4(d)**, as observed in both biplot components. In a multivariate environment especially via PCA, a selective feature-based approach has demonstrated the potential for further classification to take place effectively, by selecting variant parameters of the data as shown in **Fig** **S4(c)**.

1. **Gender Analysis: Another normalization factor**

**Fig S5** shows the discriminant analysis scores and canonical plot on classifying gender from the raw data, augmented with the feature-extracted data which is independent of the other normalized spectra. Refer **S1 Dataset**.


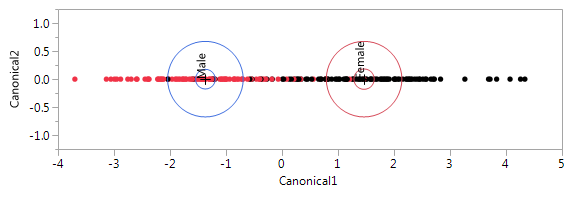


**Fig S5**. Discriminant Analysis on gender

Based on the statistical model from the discriminant analysis, a 93.5% prediction accuracy was obtained. This renders gender as playing a partial role in the spectrum, hence a normalization factor. In previous studies, however, the findings were not conclusive towards the capability of spectroscopic techniques in discriminating gender, but only in terms of spectroscopically-derived reactive hyperemia (RH) levels versus gender. It was reported that males reported higher RH levels when exposed to ischemic load [[12](#_ENREF_12)]. We consider this particular finding novel in its own merit, based on a directly measured diffuse reflectance spectroscopy, without any derivation of any skin-related measurements. Future investigations to verify these findings are solicited.

**References**

1. Nogueira MS, Raju M, Gunther J, Grygoryev K, Komolibus K, Lu H, et al., editors. Diffuse reflectance spectroscopy for determination of optical properties and chromophore concentrations of mice internal organs in the range of 350 nm to 1860 nm. Biophotonics: Photonic Solutions for Better Health Care VI; 2018: International Society for Optics and Photonics.

2. Lim L, Nichols BS, Rajaram N, Tunnell JW. Probe pressure effects on human skin diffuse reflectance and fluorescence spectroscopy measurements. Journal of biomedical optics. 2011;16(1):011012.

3. Nath A, Rivoire K, Chang SK, Cox DD, Atkinson EN, Follen M, et al. Effect of probe pressure on cervical fluorescence spectroscopy measurements. Journal of biomedical optics. 2004;9(3):523-34.

4. Tan TY, Zhang L, Neoh SC, Lim CP. Intelligent Skin Cancer Detection Using Enhanced Particle Swarm Optimization. Knowledge-Based Systems. 2018.

5. Matas A, Sowa MG, Taylor G, Mantsch HH. Melanin as a confounding factor in near infrared spectroscopy of skin. Vibrational spectroscopy. 2002;28(1):45-52.

6. Hani A, Nugroho H, Noor NM, Rahim K, Baba R, editors. A Modified Beer-Lambert Model of Skin Diffuse Reflectance for the Determination of Melanin Pigments. 5th Kuala Lumpur International Conference on Biomedical Engineering 2011; 2011: Springer.

7. Sharma V, Gupta V, Jangid B, Pathak M. Modification of the Fitzpatrick system of skin phototype classification for the Indian population, and its correlation with narrowband diffuse reflectance spectrophotometry. Clinical and experimental dermatology. 2018.

8. See JA, Goh CL, Hayashi N, Suh DH, Casintahan FA. Optimizing the use of topical retinoids in Asian acne patients. The Journal of dermatology. 2018;45(5):522-8.

9. Silpa-archa N, Kohli I, Chaowattanapanit S, Lim HW, Hamzavi I. Postinflammatory hyperpigmentation: A comprehensive overview: Epidemiology, pathogenesis, clinical presentation, and noninvasive assessment technique. Journal of the American Academy of Dermatology. 2017;77(4):591-605.

10. Schaafsma W. 40 Selecting variables in discriminant analysis for improving upon classical procedures. Handbook of Statistics. 1982;2:857-81.

11. Cooksey CC, Tsai BK, Allen DW, editors. Spectral reflectance variability of skin and attributing factors. Radar Sensor Technology XIX; and Active and Passive Signatures VI; 2015: International Society for Optics and Photonics.

12. Sprigle S, Linden M, Riordan B. Characterizing reactive hyperemia via tissue reflectance spectroscopy in response to an ischemic load across gender, age, skin pigmentation and diabetes. Medical engineering & physics. 2002;24(10):651-61.
